# Molecular targets and system biology approaches for drug repurposing against SARS-CoV-2

**DOI:** 10.1186/s42269-020-00444-3

**Published:** 2020-11-19

**Authors:** Rahul Kunwar Singh, Brijesh Singh Yadav, Tribhuvan Mohan Mohapatra

**Affiliations:** 1 Department of Microbiology School of Life Sciences, H.N.B. Garhwal University, Srinagar (Garhwal), Uttarakhand 246174 India; 2 Faculty of Biosciences and Aquaculture, Nord University, 8026 Bodo, Norway; 3 Department of Microbiology, Institute of Medical Sciences, Banaras Hindu University, Varanasi, 221005 India

**Keywords:** COVID-19, Drug targets, Drug repurposing, Interaction network, Network biology

## Abstract

**Background:**

COVID-19, a pandemic declared by WHO, has infected about 39.5 million and killed about 1.1 million people throughout the world. There is the urgent need of more studies to identify the novel drug targets and the drug candidates against it to handle the situation.

**Main body:**

To virtually screen various drugs against SARS-CoV-2, the scientists need the detail information about the various drug targets identified till date. The present review provides the information about almost all the drug targets, including structural and non-structural proteins of virus as well as host cell surface receptors, that can be used for virtual screening of drugs. Moreover, this review also focuses on the different network analysis tools that have been used for the identification of new drug targets and candidate repurposable drugs against SARS-CoV-2.

**Conclusion:**

This review provides important insights of various drug targets and the network analysis tools to young bioinformaticians and will help in creating pace to the drug repurposing strategy for COVID-19 disease.

## Background

SARS-CoV-2, as named by International Committee on Taxonomy of Viruses (ICTV) on February 11, 2020, is a novel viral pathogen and is responsible for the ongoing COVID-19 pandemic. The pandemic is thought to be originated from Wuhan city of China in December 2019 (Perlman 2020) and has caused mortality of about 1.1 million people throughout the world. This pathogen belongs to the genus beta-coronavirus of the family coronaviridae, a family of enveloped, positive-sense single-stranded RNA virus (Yang and Wang 2020). The virus is round or oval in shape, with a diameter of approximately 60–140 nm and revealed a crown-like appearance under an electron microscope (Zhu et al. 2020). The spread of this new virus has been faster than the other known human coronaviruses. After the identification of this novel pathogen, its first genome sequence was made available in the public domain by the Chinese scientists in January 2020 (Ren et al. 2020). The genome sequence revealed that this novel virus has only 80% similarity with the SARS-CoV-1 which was responsible for SARS epidemic in 2002 and about 96% similarity with the bat coronavirus, Bat-Cov RaTG13 (Wu et al. 2020). Based on the genome sequence similarity with RaTG13, bats could be the primary reservoir for SARS-CoV-2. After that, almost every country affected with this pandemic has sequenced the genome of this novel pathogen. Comparative analysis of these whole genome sequences has identified total 116 mutations among the viral genome till date (Khailany et al. 2020). Recently, three different strains of this novel pathogen, based on the mutations in its genome, have been identified in the patients from across the world using phylogenetic network analysis (Forster et al. 2020). All the sequence information of SARS-CoV-2 is available free of cost at NCBI database, allowing the global medical and scientific community to rapidly design and deploy molecular diagnostics and the drug candidates against the novel pathogen.

Despite the free availability of the whole genome sequence, and lot of efforts being made for the drug discovery/ repurposing, there has been no vaccine available for use to fight against the COVID-19 pandemic and only few antiviral drug available for treatment of mild and moderate cases only. The risk of re-infection/ reactivation of this pathogen and the lack of appearance of symptoms in re-infected patients are posing a more serious threat to humanity. This situation demands an urgent need for the development of effective and safe therapeutic medicine and vaccines (Tai et al. 2020). Currently, the researchers across the world are trying to either repurpose the molecules already available in databases or develop new drugs against COVID-19 based on the therapeutic targets predicted from the knowledge about the genome and pathogenesis of SARS-CoV-2. The system and network biology approaches are offering the identification of novel drug targets and repurposable drug candidates in the limited time. For screening of various drugs available in databases, as well as using network biology approaches for identification of new targets, the scientists need the information about already identified drug targets. Therefore, this review summarizes all the therapeutic targets identified to date and the system biology-based approaches used for drug repurposing against this novel pathogen. This will be helpful in creating a pace to the researches on drug repurposing/development.

## Methodology

Four different databases, namely Nature, PubMed, ScienceDirect and Google Scholar, were searched using the keywords, drug target, drug repurposing, SARS-CoV-2 and system biology. The papers published in English language only were used for preparing this review.

## Main text

### Genome of SARS-CoV-2

**Fig. 1:**
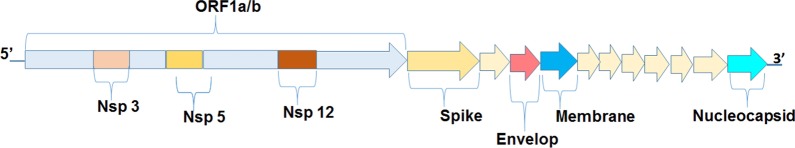
Genome organization of SARS-CoV-2

The genome of SARS-CoV-2 is a positive-strand single-stranded RNA made up of 29,891 nucleotides encoding 9860 amino acids. It consists of a 5′-terminal untranslated region (UTR), twelve functional open reading frames (ORFs) coding for structural and non-structural proteins and a -3′UTR (Fig. 1). Out of 12 ORFs, the first ORF covering almost two-thirds of viral genome translates into a polyprotein, pp1ab and cleaves into 16 non-structural proteins. The 16 non-structural proteins include two viral cysteine proteases, namely NSP3 (papain-like protease PLpro), NSP5 (chymotrypsin-like main protease 3CL pro), NSP10/16 (RNA methyltransferase), NSP12 (RNA-dependent RNA polymerase, NSP13 (helicase) and other NSPs which are likely to be involved in the transcription and replication of the virus. At the downstream of the first ORF, other ORFs encoding structural proteins such as S (spike), E (envelope), M (membrane) and N (nucleotide), interspersed with the accessory ORFs essential for in vivo pathogenesis, are present (Mousavizadeh and Ghasemi 2020; Lu et al. 2020).

The structural protein, S, specifically binds to the receptor of the host cell, and this is the key protein for viruses to enter the susceptible cells. The M and E proteins are involved in the formation of the virus envelope, while the N protein is involved in the assembly of the virus (Yang and Wang 2020).

## Drug targets

Several workers have conducted bioinformatics study on the proteins encoded by SARS-CoV-2 genome as well as the host proteins to identify the therapeutic targets and submitted their 3D model in protein data bank (PDB). The PDB ID of some of these proteins is given in Table 1. Based on their functions, these proteins can be categorized as: (1) proteins helpful in viral replication, (2) structural proteins helpful in binding with human host, (3) virulence proteins (4) host-specific enzymes.

**Table 1:** Potent target proteins with available 3D models and their PDB ID for drug development against SARS-CoV-2

S	Name of protein	Origin	PDB (protein data bank) ID
1	ACE-2	Host	6M0J
2	Apo-carbonic anhydrase II	Host	6LUV
3	3CLPro	Viral	6Y84
4	Extracellular portion of CD147	Host	3B5H
5	Furin	Host	1P8J
6	Nsp12-NSP7- NSP8 complex	Viral	7BW4
7	PLPro	Viral	4MM3
8	RDRp	Viral	6M71
9	S-Protein	Viral	6VXX, 6LZG

### Proteins helpful in viral replication

Non-structural proteins (NSPs) encoded by ORF1a/b of the SARS-CoV-2 genome are involved in various essential functions like RNA replication, transcription, translation, processing, modification and infection of the host. Many of these proteins, RdRp, helicase, 3CLpro and PLpro, are the most important targets for the development of small-molecule inhibitors due to their known biological functions and vital enzyme active site (Wu et al. 2020).

#### RNA-dependent RNA polymerase (RdRp)

RNA-dependent RNA polymerase (RdRp), also known as Nsp12, is an essential enzyme required for viral replication and transcription and has a conserved Ser-Asp-Asp domain (Huang et al. 2020). The synthesis of RdRp RNA requires a de novo synthesized 6 nucleotides long primer encoded by NSP8. Moreover, the binding of Nsp12 to RNA and its enzyme activity is enhanced by the Nsp7-Nsp8 complex. The Nsp12 core protein is a single chain of about 900 amino acids and can be divided in two domains, a N-terminal and a polymerase domain. Like other polymerases, the polymerase domain of Nsp12 adopts a cup shaped right hand structure and is comprised of finger, thumb and palm subdomains (Mirza et al. 2020). Similar to RdRp of other coronaviruses, finger and thumb subdomains of SARS-CoV-2 Nsp12 interact with each other creating an active site in the center for substrate entry through a template. Besides, the active site also has seven conserved motifs which help in binding of substrate with the template and its catalysis (Mirza and Foreyen 2020; Peersen 2017). The stability to 3D structure of the Nsp12 protein is provided by two zinc ions, one of which binds with His295, Cys301, Cys306 and Cys310 residues in the N-terminal domain whereas another binds with Cys487, His642, Cys645 and Cys646 residues in finger subdomain of polymerase domain. As these ions are situated far from the active site, they have no direct role in the polymerase activity of the protein (Ahmad et al. 2020; Kirchdoerfer and Ward 2019).

The RdRP protein has been used as significant target for drug discovery against SARS-CoV and MERS-CoV also. In principle, targeted inhibition of Nsp12-RdRp could not cause significant toxicity and side effects on host cells. Recently, ribavirin, remdesivir, sofosbuvir, galidesivir and tenofovir have been considered as potent drugs against SARS-CoV-2 since they tightly bind to its RdRp (Elfiky 2020). Out of these drugs, remdesivir has already been approved for clinical use in mild and moderate cases of COVID-19. In addition, the GTP and other nucleotide analogs may also be tested against this protein.

#### Helicase

SARS-CoV-2 Helicase, also called as Nsp13, is a multi-function protein of 596 amino acids that have five domains including two RecA like domains (1A & 2A), 1B domain, a N-terminal Zn^2+^ binding domain (ZBD) and a stalk domain connecting 1B and ZBD. These domains collectively form a triangular pyramid shaped structure. This protein has been an indispensable element for the replication of SARS-CoV-2 (Mirza and Foreyen 2020). Habtemarium et al. (2020) have analyzed the role of SARS-CoV-2 helicase (Nsp13) in viral replication as well as its structural and functional similarity with that of the helicase enzyme in other coronaviruses. They have concluded that the sequences of helicase (Nsp13) enzyme are conserved among coronaviruses and thus provide a potential target for screening of antiviral drugs.

Shu et al. (2020) have tested the biochemical activities of recombinant nsp13 of SARS-CoV-2 and showed that the protein also has the NTPase activities with ability to hydrolyze all NTPs. The energy released by hydrolyzing the NTPs is used for unwinding the RNA helices. Jang et al. (2020) have also realized the requirement of ATP hydrolysis for the helicase activity of SARS CoV Nsp13. Besides, the enzyme has a conserved NTPase active site residues, Lys288, Ser289, Asp374, Glu375, Gln404 and Arg567 as observed in SARS-Nsp13 too (Mirza and Foreyen 2020). Thus, the strategies to target the nsp13 activity include inhibition of NTPase activity, blockage of ATP/nucleotide binding to the helicase, and inhibition of helicase translocation (Habtemariam et al. 2020). Despite the promise for inhibition of Nsp13 in coronaviruses by different compounds like imidazole, quinolone, anthracycline, etc., only few reports are there about Nsp13 inhibitors for SARS-CoV-2 (Wu et al. 2020). Thus, the area demands the attention of researchers.

#### Papain-like proteinase (PLpro)

PLpro is a part of Nsp3, a 213-kDa multi-domain membrane-associated polypeptide, along with a phosphatase, a transmembrane domain, a conserved acidic domain and a Y domain. Ratia et al. (2006) have determined the structure of 35-kDa catalytic domain of SARS CoV PLpro (a.a. 1541–1855) and concluded that the protein has four distinct domains including a 62 amino acid independent N-terminal Ubl domain which makes a grip fold. Rest of the protein make a prolonged right-hand architecture with different thumb, finger and palm domains. Besides, the bioinformatics and biochemical analysis have shown the existence of a zinc binding domain in PLpro. The mutational analysis of cysteine amino acids in PLpro that binds with Zn has proved that these are required for the protease activity as well as the structure maintenance of the protein. Similar to the papain like proteases of other viruses, the active site of SARS CoV-2 PLpro also has a catalytic triad of Cys, His, Asp. The catalytic Cys112 and His273 are 3.7 Å apart from each other and are located at the base of thumb and palm domain, respectively. However, the Asp287 is located within 2.7A situated in a classic triad formation, within hydrogen-bonding distance (2.7 Å) of His273.

PLpro cleaves the replicase poly-protein (PP1a/b) to release Nsp1 and Nsp2, which is essential for ensuring correct viral replication. This protein has also showed a significant role in evading the host’s innate immunity. Being the necessary stakeholder in the process of replication in SARS-CoV-2 and other coronaviruses and their infection in host, Nsp3 has been recognized as a common target for coronavirus inhibitors. It is very valuable for targeting PLpro to treat coronavirus infections, but no inhibitor has been approved by the FDA till date. The anti-virus drugs like ribavirin, valganciclovir and thymidine may have high binding affinity to PLpro (Elfiky 2020) and need thorough investigations.

#### 3C-like main protease (3CLpro)

The 3CLpro, also known as Nsp5, of SARS-CoV-2 contains 306 amino acids. The active site of this enzyme is situated in a cleft between domain I and domain II, similar to the 3CLpro of SARS-CoV. The active site has a catalytic dyad, His41–Cys145, including one residue from each domain connected to the helical domain III by a long loop (Mirza and Foreyen 2020). Domain III, a globular cluster of five helices, is involved in regulation of dimerization which is required for the catalytic activity of the enzyme (Zhang et al. 2020).

3CLpro is first protein, cleaved automatically from replicase poly-protein (PP1a/b), to produce mature enzymes, and then further cleaves the polyprotein downstream at 11 sites to generate Nsp4–Nsp16 (Jin et al. 2020). Nsp 5 directly arbitrates the maturation of NSPs, which is essential in the life cycle of the virus.

Inhibitors targeting at Nsp5 of SARS-CoV mainly include peptide inhibitors and small-molecule inhibitors. The anti-asthmatic drug montelukast showed low binding energy to this particular proteinase it fits properly into the active pocket of SARS-CoV-2 Nsp 5. Recently, the organoselenium compound, ebselen, also unveiled promising antiviral activity in cell-based assays (Jin et al. 2020). The thorough study on the structure and catalytic mechanism of Nsp5 makes it a striking target for anti-coronavirus drug development (Zhang et al. 2020).

#### RNA methyltransferase

Recently, Feinberg School of Medicine, Northwestern University, has mapped the atomic structure of two critical proteins, Nsp10/16 (Kramer and Paul 2020; Kadioglu et al. 2020). Nsp16 has 2′-O-RNA methyltransferase activity and modifies the genetic material of the SARS-CoV-2 by RNA cap formation. The capping of viral RNA provides it a look more like the host (human) cell RNA thereby allowing it to hide from the cell’s defense system. This offers the stability and time for multiplication of viral RNA. The Nsp10 acts as cofactor for the Nsp16 activity. If a drug can be developed to inhibit Nsp10/nsp16, the immune system would be able to detect the virus and eradicate it faster. The active site of Nsp16 is highly conserved between the SARS CoV-and SARS-CoV-2 suggesting the feasibility for development of methyltransferase inhibitors against several coronaviruses. One such inhibitor, sinefungin, and its binding capacity with Nsp10/16 complex has been explained recently by Krafcikova et al. (2020).

#### Other proteins

Besides the above targets, some other non-structural proteins, including Nsp7-Nsp8 (primase) complex, Nsp9, Nsp14 (exoribonuclease) and Nsp15 (endonuclease), also play an important role in the virus RNA synthesis and replication, suggesting that these proteins may be useful targets for the anti-viral drug discovery. Structures of Nsp15 endonuclease and Nsp9 replicase have recently been determined by the University of Chicago (Kramer and Paul 2020).

### Structural proteins helpful in binding with human host

#### Spike protein

**Fig. 2:**
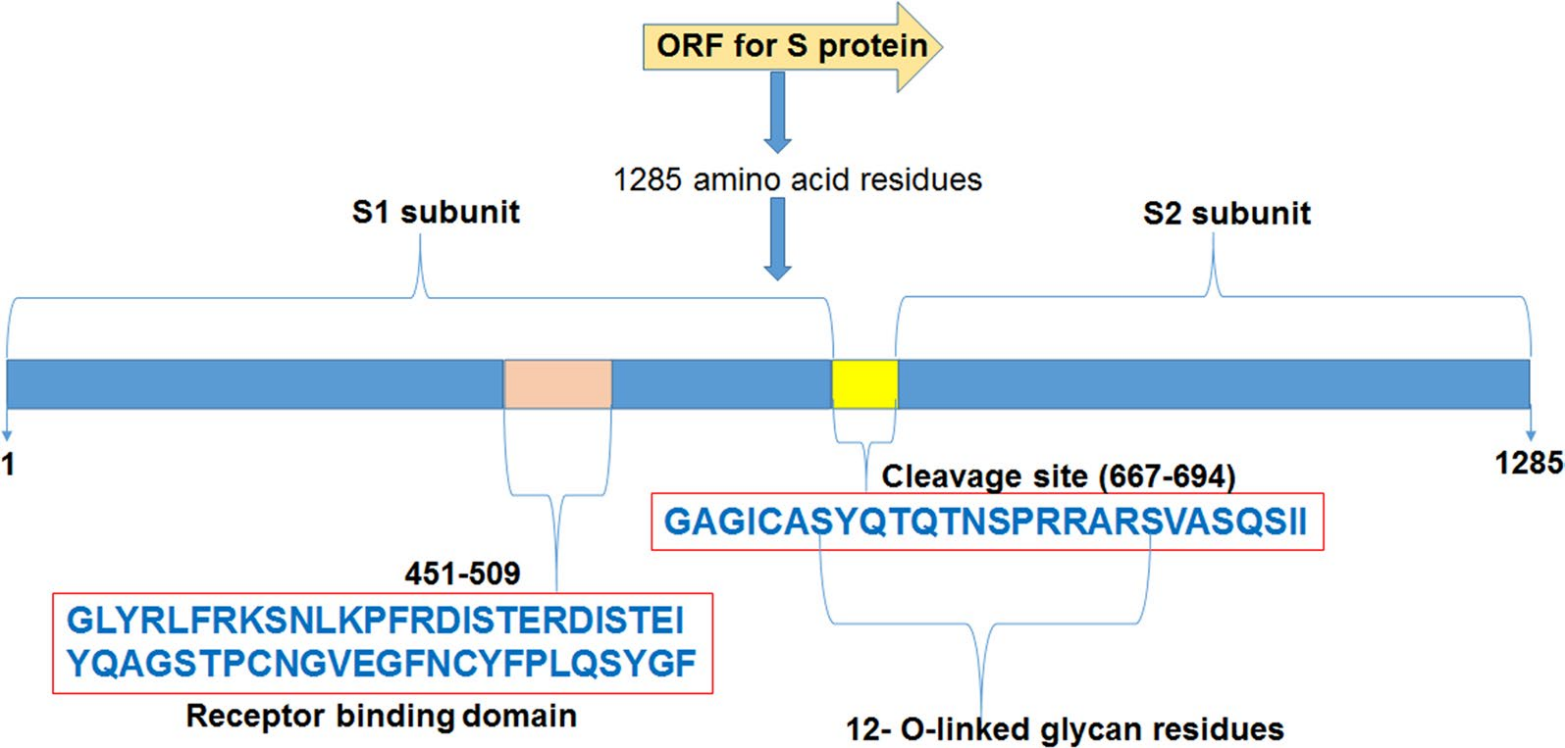
Features of SARS-CoV-2 spike protein along with amino acid sequences of receptor binding domain and the cleavage site

Spike (S) proteins of SARS-CoV-2 are present on the surface of the virus as a trimer and have a receptor binding domain (RBD) for ACE-2 (angiotensin converting enzyme 2) receptor protein present on the surface of cells of human respiratory system. These S proteins promote the viral invasion into host’s cells and are the main goal of host’s immune system too. Thus, these proteins offer an opportunity for the designing of antiviral drugs and vaccines inhibiting the entry of the virus (Walls et al. 2020). The spike protein of SARSCoV-2 has a functional polybasic (furin) cleavage site of 28 nucleotides at the S1-S2 boundary (Andersen et al. 2020). This cleavage site is also characterized by the presence of 12 O-linked glycan residues (Fig. 2). The presence of this cleavage site is a notable feature of SARS-CoV-2 and is identified by the host’s serine protease TMPRSS-2 for S protein priming. A small peptide molecule that can bind on this cleavage site may inhibit the S protein priming and thereby could inhibit the viral entry in the host cell.

#### Envelope protein

Envelope (E) protein has a vital role in assembly and morphogenesis of SARS-CoV-2. It possesses important biological functions for the structural integrity of coronavirus and its virulence in host. It is also known to activate the NLRP3 inflammasome of host, which leads to the overproduction of IL-1beta interleukin. This protein may be one of the possible targets of chloroquine.

### Virulence proteins

The non-structural viral proteins Nsp1, Nsp3c and ORF7a are known to impede with innate immune system of host and help the virus to hide and are called virulence proteins. Nsp1 promotes the degradation of host mRNA and prohibit the production of antiviral interferons by binding with the 40S ribosomal subunit of host cell. The protein Nsp3c binds with ADP-ribose of host and helps the virus to struggle with host’s innate immunity. The protein encoded by ORF7a is helpful in release of newly assembled virions as it binds with bone marrow matrix antigen 2 (BST-2). The binding of ORF7 with BST-2 inhibits its glycosylation and eventually its activity, i.e., the inhibition of viral release (Wu et al. 2020). Thus, these virulence proteins may be attractive targets for drug discovery against SARS-CoV-2.

### Host-specific enzymes

#### Angiotensin converting enzyme II (ACE-2)

**Fig. 3:**
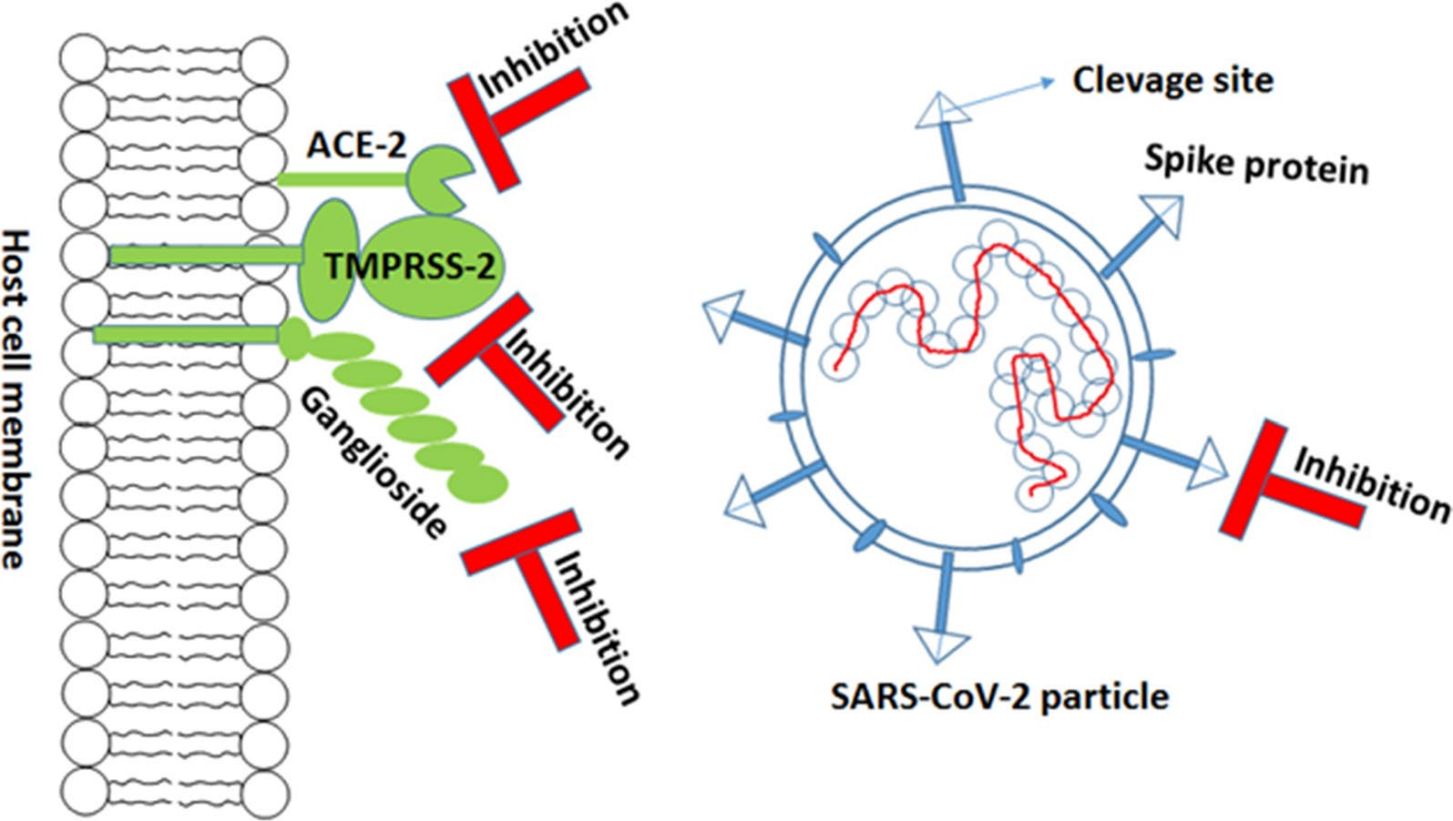
Host cell surface receptors helpful in entry of SARS-CoV-2 in host cell

The novel coronavirus, SARS-CoV-2, invades the human cells by receptor mediated endocytosis using the cell surface receptor protein angiotensin converting enzyme II (ACE-2). This host-specific enzyme has been proved a specific receptor for binding with the receptor binding domain of viral spike (S) protein in case of both SARS-CoV and SARS-CoV-2 (Hoffmann et al. 2020; Wu et al. 2020). Six amino acids, L455, F486, Q493, S494, N501 and Y505, present in the receptor binding domain of SARS-CoV-2 S protein are critical for binding with ACE2 receptors. Out of these six amino acid residues, five differ from that in the SARS-CoV (Andersen et al. 2020). Thus, ACE-2 is a crucial target for antiviral drugs to prevent the entry of novel coronavirus into the host cell (Fig. 3). Natural antibodies from convalescent plasma as well as the engineered antibodies may be used for the purpose. However, much research is needed to know the status of ACE2 expression in various tissues of COVID-19 patients after the administration of such inhibitors in COVID-19 (Li et al. 2020a; b). Chloroquine is reported to interrupt the interaction between viral S protein and ACE2 by inhibiting the glycosylation of ACE2 (Fantini et al. 2020). Further, drug-repurposing strategies have been suggested to find possible molecule that may bind to S-protein to disrupt S protein-ACE2 interaction (Letko et al. 2020).

#### TMPRSS-2

The phenomenon of binding of viral spike (S) protein with ACE-2 requires S protein priming, i.e., the cleavage of S protein into two subunits, S1 and S2, through a serine protease enzyme of host origin, TMPRSSS-2 (Hoffman et al. 2020). This protease enzyme is highly expressed in goblet and ciliated cells of nasal epithelium (Sungnak et al. 2020). The S1 subunit of viral spike protein binds with the ACE-2, whereas the S2 subunit helps in the fusion between the viral and host cell membrane. The strategy to block the activity of TMPRSS-2 may be a promising tool for the development of antiviral drug against SARS-CoV-2. The whole structure of this serine protease enzyme measures 42 × 24 Å and consists of an N-terminal activation domain and a C-terminal catalytic domain. Vishnubhotla et al. (2020) have mapped the active site of the enzyme and noticed the highly conserved nature of amino acid residues His296, Ser441, Lys432, Trp461 and Gln438 present in this site.

Hoffman et al. (2020) have shown that inhibitors of this serine protease can block the entry of SARS-CoV-2 in the host cell. The use of a known serine protease inhibitor, camostat mesilate, has shown the 65% reduction in mortality of mouse infected with SARS-Co-V (ClinicalTrials.gov Identifier: NCT04321096). Thus, the clinically proven serine protease inhibitors may be tested as antiviral medicine against the SARS-COV-2 (Hoffman et al. 2020).

#### Gangliosides

Recently, a new domain, conserved among clinical isolates of SARS CoV-2, has been identified at the N-terminus of S protein. This domain binds with the gangliosides on the host cell surface to facilitate the contact of viral S1 subunit with the host’s ACE-2 receptor (Fantini et al. 2020). Recently, it has been shown that the chloroquine and its derivative, hydroxychloroquine, binds efficiently with the sialic acids present on gangliosides and thus blocks the binding of the viral S protein with the gangliosides (Fantini et al. 2020).

#### Basigin (BSG)

In addition to ACE-2, another host cell surface glycoprotein receptor molecule, BSG may also mediate the invasion of SARS-CoV-2 into host cell (Ulrich and Pillat 2020). This molecule, also known as CD147 or extracellular matrix metalloproteinase inducer (EMMPRIN), also provides a specific binding site for receptor binding domain of viral spike (S) protein. It has been observed that blockage of CD147 receptor using meplazumab, an anti-CD147 humanized antibody, inhibited the invasion of virus into host cell (Wang et al. 2020). A phase II clinical trial is currently underway in China investigating the efficiency of meplazumab injection to treat the pneumonia caused by SARS-CoV-2 (ClinicalTrials.gov Identifier: NCT04275245).

## Systems biology-based approaches for identification of targets and repurposable drug candidates against SARS-CoV-2

The Covid-19 disease spreading speed is very fast throughout the world; the traditional approaches to control the disease are not feasible at this moment. The alternate and convincing strategy is to recognize the already approved drugs that can be repurposed against the disease.

The main strategy in system biology-based method is the designing of multiple networks which demonstrate every level of omics spectrum and their assimilation in a layered network that correlate information within and between layers (Oulas et al. 2019). The system-based molecular networks of the available repurposable drug candidates with their potential targets provide the complete information about the genes expressed, the encoded proteins and metabolites involved during progression of the stress (Yadav et al. 2016). The data used for the genomics, proteomics and metabolomics analysis of the disease are either retrieved from the available literature or obtained from next generation sequencing (NGS) and genome-wide association sequencing (GWAS) analyses (Yadav et al. 2017). Contrary to the virtual screening approach, in which targets are already known from the beginning, system biology has capability to predict unknown targets in the genome and metabolome of the pathogen (Yadav and Tripathi 2018). Since the last decade, plenty of computational tools based on system biology have been developed and validated (Cheng et al. 2018; Guney et al. 2016; Zitnik et al. 2018; Zhou et al. 2020a). These tools enhanced the speed of therapeutics and diagnostics process.

### The system network-based drug repurposing tactics

The system-based network drug repurposing methodology designated in this section is that the drugs have the potential to perturb the network vicinity of the virus disease module. There are two important target proteins in human for COVID-19, angiotensin-converting enzyme-2 (ACE2) and basigin (BSG); therefore, researchers in the area of system biology utilize these proteins to make host–pathogen interactions network with high confidence. The human interactome with SARS-CoV-2 to build an important sub-network using ACE2 and BSG targets. The host (human) COVID-19 infection PPI (protein–protein interaction) network was constructed which focus the SARS-CoV-2 spike protein receptors. The receptors ACE2 and BSG were considered as seed nodes for the execution of random walk with restart (RWR) algorithm. The sub-network of protein–protein interaction showed influence of corona invasion on lethal comorbidities via insulin resistance. The network provides the significance of the molecular basis of COVID-19 fatality with relation of AGE-RAGE (receptor for advanced glycation endproducts) signaling pathway in diabetic complications and adipocytokine signaling pathway. Further, the identification of drug molecule, which potentially downregulates ACE2 with other critical proteins, was also identified with network-based approach. This analysis recognized three drug molecules incyclinide (also known as COL-3), entinostat and mocetinostat, which show activity against corona disease, and they can be repurposed for COVID-19 (Chakrabarty et al. 2020; Beck et al. 2020).

The interactions network of host’s protein with viral protein provides detail information related to structural and functional domain of a particular site of infection that further helps to create drug–target interactions network. User-friendly internet-based interface was developed to represent a visual presentation of the virus–human interactome utilizing network-based algorithms for prediction of drugs named as CoVex. The CoVex network delivers information about the drug targets based on experimentally validated interaction of all the known human target proteins with SARS-CoV-2 and SARS-CoV-1 proteins. The CoVex interface also reconnoiters the molecular mechanisms of the virus life cycle and predict repurposable drug candidates involved in these mechanisms (Sadegh et al. 2020). The major focus of the network-based toolset is to improve the basic respiratory manifestations of the virus in the lung and detected comorbidities related with cardiovascular diseases.

### Network proximity, diffusion and AI (artificial intelligence)-based metrics

The main aim of system biology is to identify important regions in the network that can be linked to biological functions with pathologies. The network diffusion-based method elucidates all kind of omics in references to molecular interaction. The network proximity is defined through the network smoothing index, which allow to calculate number of omics information in genes in their neighborhoods network utilizing network diffusion (Bersanelli et al. 2016). In the present big data era, artificial neural network (ANN) offers innovative technique of information science to describe diseases, medicines, therapeutics and characterized targets with minimum errors. The artificial intelligence (AI) potentially reduces timelines and overall costs for medicine technologies and therapeutic development. In AI-based network medicine, the main idea is to screen the libraries of already existing drugs with numerous tests that might reveal new applications, and have made understandings that how to manage the medicines designed for one disease to treat another one (Zhou et al. 2020b). The AI and network medicine method for a drug repurposing strategy show a broad picture of molecular landscape of the SARS-CoV-2 infection based on prior information like experimentally validated human proteins recognized as viral interactors, tissue-specific gene expression data. The network investigated the host molecular interaction network to the COVID-19 invasion via propagation and connectivity significance to conclude to propose drugs for the treatment of COVID-19. The potential target genes were identified based on ranking and coherent groups of tissue, potential and distinct sites of interactions between the virus and the organism (Stolfi et al. 2020).

A complex approach was established to utilize the three network-based drug repurposing tactics, namely network proximity, diffusion, and AI (artificial intelligence)-based metrics. The network found that virus could affect respiratory system as well as other tissues like reproductive system, brain regions and neurological comorbidities. Moreover, this approach identified 81 promising drug candidates for repurposing against COVID-19 and ranked them based on their expected efficacy (Gysi et al. 2020).

The systematic investigation on interaction of human proteins with SARS-CoV-2 during infection was also accomplished. In this study, 27 different SARS-CoV-2 genes were cloned and expressed into human HEK293T cells as 2xStrep-tag fusion proteins. Later, these tagged viral proteins were isolated and examined for their association with human proteins using affinity-purification-mass spectrometry technique. The study recognized the major target proteins including lipoprotein metabolism, nuclear transport and ribonucleoprotein, followed by the possible binding interfaces. To the end, a network study showed 332 human proteins interacting with SARS-CoV-2 viral genes and developed a first interactome model for SARS-CoV-2 and human protein interactions (Gordon et al. 2020). Still at present, scientific community struggling with proper knowledge of the molecular basis of SARS-CoV-2 disease prevents a complete assessment of small lead candidates for host-directed therapies. The system and network-based investigation mapped the interaction landscape between SARS-CoV-2 proteins and human proteins. Consequently, a network model comprising of COVID-19 disease with 1,344 genes utilized an iterative network-building algorithm called AutoNet. The genome sequence analysis of SARS-CoV-2 was performed, which reveled disease similarities with SARS, MERS and other human coronavirus. The network of COVID-19 showed 34 related genes, including ACE2 and 24 enriched pathways in five topological network modules. The scanning of this network with already known drug target interactions offered 78 drugs repurposable against COVID-2019. To conclude, based on action mechanisms and adverse effects of the drugs, the study has recommended 30 drugs for clinical approval (Li et al. 2020a; b).

**Fig. 4:**
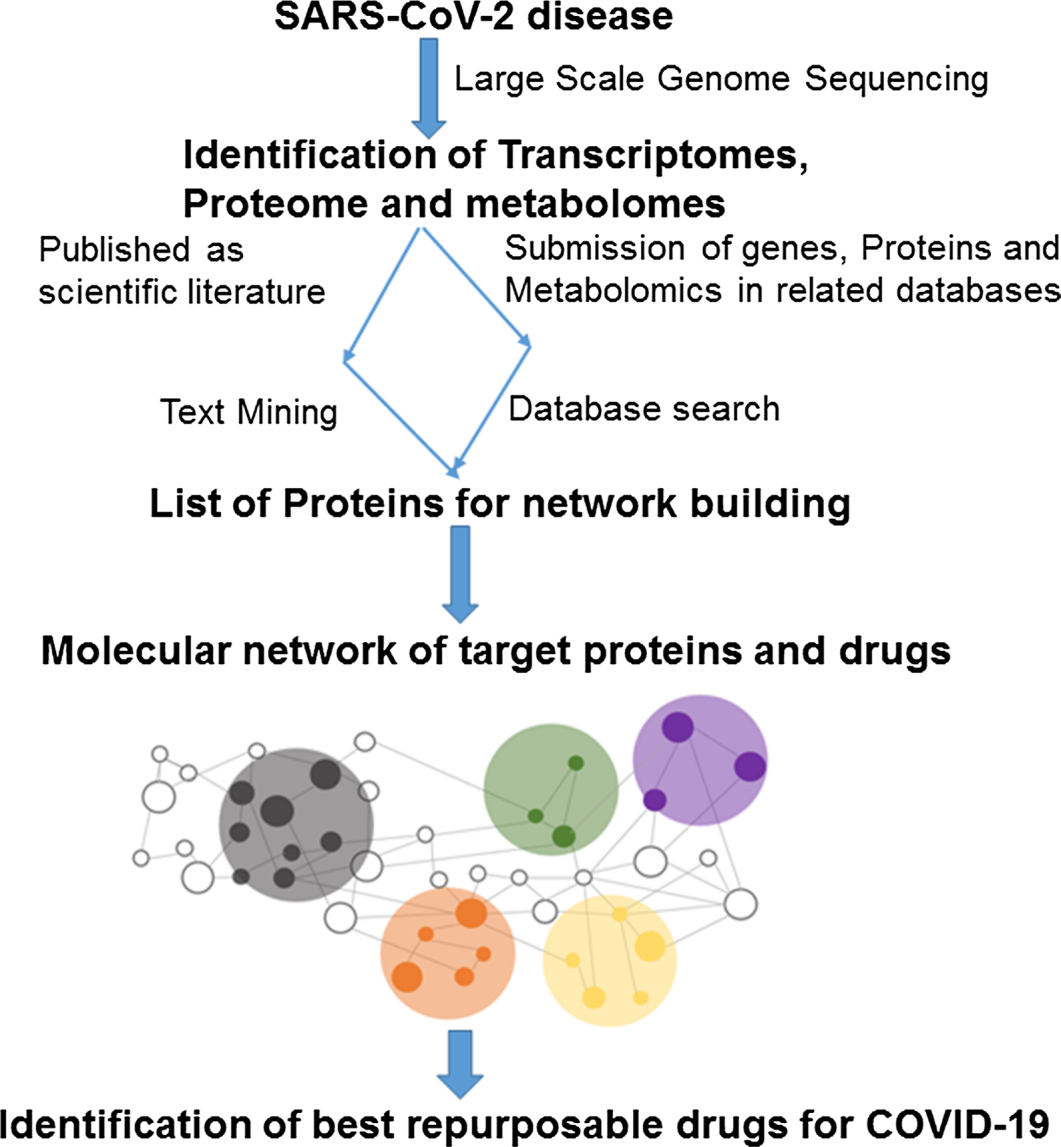
Diagrammatic representation of system biology-based methods for identification of therapeutic targets and repurposable drugs against SARS-Co-2

Moreover, an influential network-based approach was also used for rapid identification of candidate repurposable drugs and potential drug combinations against SARS-CoV-2. This method utilized the system pharmacology-based network platform to explore the interaction between SARS-CoV-2–host interactome and drug targets in the human protein–protein interaction network. The network presented 16 potential drugs candidates repurposable against SARS-CoV-2 with known targets and further validated those using enrichment analyses of drug–gene signatures and the virus-induced human cell line transcriptome. After validation, three potential drug combinations were recommended for repurposing (Zhou et al. 2020b). Thus, the biological network-based approaches are very useful for discovery of new repurposable drugs candidates and their targets identification (Fig. 4). The drug repurposing may produce new therapies at a quicker rate than novel drug discovery when the safety profiles of the drugs being repurposed have been evaluated in the context of drug development for another disease, and at an even faster rate when the drugs have been approved for other diseases and marketed. Some repurposable drug candidates, against COVID-19, identified through system and network biology given in Table 2.

**Table 2:** Network-predicted repurposable drugs with anti SARS-CoV-2 evidence ( adopted from Zhou et al. 2020a, b)

S. No	Drug name	Chemical structure	Known as	PubMed ID
1	Camphor		Antipruritic, anti-infective	27833881
2	Colchicine		Anti-inflammatory	28795759
3	Emodin		Anti-inflammatory	21050882
4	Eplerenone		Anabolic steroids	12815555
5	Equilin		Estrogen	27169275
6	Melatonin		Hormone	25262626
7	Mercaptopurine		Antimetabolites, antineoplastic	18313035
8	Quinacrine		Antibacterial, anti-malaria	233301007
9	Sirolimus		Immunosuppressant	23135723
10	Toremifene		Antineoplastic	27362232

## Conclusion

In view of COVID-19 health crisis, repurposing of clinically proven medicines is one of the best choice to opt for. However, to get the better results these medicines must be first tested in silico for their efficacy against the target viral and host proteins. This initial level of screening will save the time and efforts required for the clinical trials of each drug. Availability of 3D models of the target proteins is definitely helping the researchers to test the large number of drugs at a faster pace. In addition, new drug targets for this novel pathogen are continuously being discovered using the genome sequence data and system biology tools. We appeal the bioinformatician throughout the world to identify novel drug targets using system biology and explore all the available drug databases for the possibility of their repurposing against SARS-CoV-2. This will help in not only finding the first line treatment but also in the preparation for pipeline of anti-COVID drugs.

## Data Availability

The datasets used and or analyzed during the current study are available from the corresponding author on reasonable request.

## Abbreviations

COVID19: Coronavirus disease 2019
SARS-CoV-2: Severe acute respiratory syndrome-coronavirus-2
ACE-2: Angiotensin converting enzyme-2
BSG: Basigin
RdRp: RNA-dependent RNA polymerase
Nsp: Non-structural protein
